# The Preventive Effect of Theabrownin from Ya’an Tibetan Tea Against UVB-Induced Skin Photodamage in BALB/c Mice via the MAPK/NF-κB and Nrf2 Signaling Pathways

**DOI:** 10.3390/foods14040600

**Published:** 2025-02-11

**Authors:** Jingyi Xu, Mingji Xie, Xing Liang, Peida Luo, Xinyao Yang, Jing Zhao, Jinlin Bian, Bo Sun, Qian Tang, Xiao Du, Yao Zou, Weidong Dai, Chunlei He

**Affiliations:** 1 Tea Department of College of Horticulture Science, Sichuan Agricultural University, Chengdu 611130, China; xujytea@126.com (J.X.); xiemingji@stu.sicau.edu.cn (M.X.); l026605@126.com (P.L.); y18031866381@163.com (X.Y.); zhaojingwxl1234@outlook.com (J.Z.); bianjinlin126@126.com (J.B.); bsun@sicau.edu.cn (B.S.); tangq2008@126.com (Q.T.); xiaodu@126.com (X.D.); zouyao@126.com (Y.Z.); 2 Sichuan Key Laboratory of Refined Sichuan Tea, Chengdu 611130, China; 3 Sichuan Academy of Agricultural Sciences, Chengdu 610066, China; lx15520480072@163.com; 4 Key Laboratory of Tea Biology and Resources Utilization, Tea Research Institute, Chinese Academy of Agricultural Sciences, Hangzhou 310008, China

**Keywords:** theabrownin, Tibetan tea, photodamage, antioxidant, anti-inflammation, apoptosis

## Abstract

Ya’an Tibetan tea, renowned as a mysterious tea, has been used as a traditional remedy for disease prevention among ethnic minorities in the Qinghai-Tibet Plateau region, which experiences the highest levels of UVB radiation in the world, for over 1000 years. Theabrownin (TB) from Ya’an Tibetan tea exhibits various health benefits. In this study, the preventive effects of TB on UVB-induced skin damage were investigated. The results showed that TB pretreatment significantly alleviated visible skin damage, epidermal hyperplasia, and collagen destruction in BALB/c mice. The mechanism of action involved increasing the mRNA and protein levels of Nrf2 and enhancing SOD enzyme activity, thereby reducing MDA content and improving the body’s antioxidant capacity. TB also inhibited the protein synthesis of inflammatory factors such as TNF-α, IL-1β, and IL-6, as well as the expression of NF-κB mRNA and protein, thereby reducing skin inflammation. Furthermore, it suppressed the overexpression of p38 MAPK, ERK, and AP-1 mRNA and protein, along with the downstream MMP-1 protein, to prevent collagen destruction in the skin. Additionally, TB pretreatment prevented cell apoptosis by reducing Caspase-3 overexpression. These results suggest that TB can prevent UVB-induced photodamage and exert its preventive effects in a dose-dependent manner by downregulating the MAPK/NF-κB signaling pathway while promoting the Nrf2 signaling pathway in the skin. Consequently, TB holds promising potential for future applications in skin photodamage prevention and skin health promotion.

## 1. Introduction

The skin, acting as the human body’s outermost defensive layer, serves to protect against harmful external factors, particularly UV radiation. Excessive exposure to UVB radiation (medium-wave ultraviolet, wavelength 290–320 nm) can damage the skin and impair its barrier function, leading to conditions such as erythema, edema, inflammatory pain, and even skin tumors [1,2]. In recent years, rapid industrialization and agricultural advancements, along with ozone depletion and the increasing use of artificial UV light sources, has significantly elevated human exposure to UVB radiation in daily life. Consequently, the adverse effects of UVB exposure have grown more pronounced, leading to a rising global occurrence of various skin conditions [3]. UVB primarily induces skin photodamage through mechanisms including oxidative stress, cell apoptosis, inflammatory reactions, and the degradation of collagen and elastin [4,5]. Mitigating and repairing UVB-induced skin photodamage is costly and often accompanied by significant side effects. Consequently, research on natural products for UVB protection has gained importance in preventing skin damage and related diseases [6].

**Figure 1:**
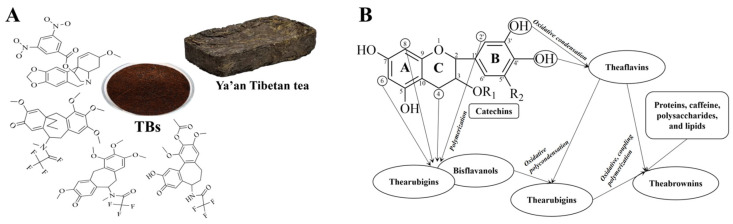
Structural characterization and formation of theabrownin (TB). (**A**) Possible structural fractions of theabrownin with different molecular weights. (**B**) The mechanism of theabrownin formation.

Theabrownin (TB) is a type of complex, water-soluble phenolic pigment. Chemically, it is a high-molecular-weight polymer formed through the oxidation of tea polyphenols into theaflavins and thearubigins, followed by further oxidation, polymerization, and binding with polysaccharides, proteins, amino acids, caffeine, and lipids (Figure 1) [7,8]. In addition to polyphenol oxidative polymers, TB contains compounds such as amino acids, sugars, hydroxyl groups, carboxyl groups, alkyl groups, and benzene ring analogs. Among these, the phenolic hydroxyl content ranges from 2.58 to 5.83 mmol/g, and the carboxyl content ranges from 0.15 to 0.68 mmol/g, representing the active groups of TB that contribute to its strong antioxidant properties [9,10]. TB, along with theaflavins and thearubigins, is the primary pigment responsible for the color and quality of fermented tea [11,12,13]. The content of TB varies among different types of tea. In general, TB constitutes 4–9% of the dry matter in black tea, serving as a major contributor to the “dull” appearance of the tea infusion [13,14]. As a key quality and bioactive component of dark tea, TB content in dark tea is significantly higher than in other types of tea, generally accounting for 7–23.68% of the dry matter [15]. Different types of dark tea exhibit varying levels of content, with Pu-erh tea containing over 13% [16], followed by Liu Bao tea (about 10%), and Ya’an Tibetan tea (>9%) [17,18]. Fu brick tea has the lowest TB content at about 7% [8]. During the processing of dark tea, tea polyphenols undergo oxidative polymerization and interact with other substances to form TB, gradually accumulating with prolonged fermentation [19]. Research indicates a substantial positive correlation between TB content and the health benefits of dark tea [18]. As a key active component in dark tea, TB exhibits various health benefits, including reducing cardiovascular risk factors, providing antioxidant properties, scavenging free radicals, and enhancing immunity [20,21,22,23]. Moreover, TB has minimal toxic side effects on the body, making its biological activity a current research hotspot in tea and natural products.

In our previous work, we established that TB in Ya’an Tibetan tea has a significant preventive effect on the antioxidant and hematopoietic systems of rats damaged by ^60^Coγ radiation [24,25,26]. Ya’an Tibetan tea, recognized as the earliest and most typical dark tea, derives its name from being produced in Sichuan’s Ya’an and sold to the Qinghai-Tibet Plateau, with a history of over 1300 years [27]. The unique processing techniques and exceptional geographical conditions of Ya’an City contribute to the distinctive quality of Ya’an Tibetan tea. Its production process has been listed as a UNESCO Intangible Cultural Heritage, namely, Dark Tea Processing Techniques VIII-152 and South Road Border Tea Processing Techniques. Ya’an Tibetan tea is made from mature fresh leaves and red moss (the tender stem of mature tea leaves near the top one-third of the branch with a purple red skin) harvested at an altitude of over 1000 m. Its processing involves eighteen steps, including four rounds of fermentation, repeated kneading, screening, tea steaming, and tea tossing. This type of tea, produced through specialized and long-lasting fermentation techniques, contains nearly 500 beneficial organic compounds, approximately 700 aromatic compounds, an abundance of TB and tea polysaccharides, and less caffeine than other teas [18]. For over 1000 years, Ya’an Tibetan tea has been the daily tea and primary prescription for disease prevention among ethnic minorities in border areas, such as the Qinghai-Tibet Plateau. The Tibetan people have a profound understanding and experience of tea, expressed in proverbs like “Three days without food, fine; one day without tea, stagnation; three days without tea, illness”. Ya’an Tibetan tea is often hailed as the “mysterious tea” due to its mellow taste, unique health benefits, and cultural significance. The high altitude of the Qinghai-Tibet Plateau makes it one of the regions with the highest UV radiation levels in the world [28,29]. However, traditional beliefs and tea-drinking practices suggest that the incidence of skin diseases among people living in high UV radiation areas is relatively low [30]. It is speculated that this may be directly related to their long-term consumption of Tibetan tea; however, no scientific evidence has yet underscored the UV radiation resistance properties of Tibetan tea or its main functional components.

In the present study, TB was extracted from Ya’an Tibetan tea, and a photodamage mouse model was constructed to evaluate the preventive effects of TB on skin photodamage through macroscopic observation and histological staining. A commercial kit was used to assess the skin and serum levels of antioxidant enzyme (SOD) activity and malondialdehyde (MDA) production in mice. The levels of the key collagenase MMP-1 and inflammatory cytokine (IL-1β, IL-6, and TNF-α) proteins during skin photodamage were analyzed using ELISA. Additionally, the expression of p38 MAPK, ERK, AP-1, NF-κB, Nrf2, and Caspase-3 mRNA and proteins in the skin was evaluated using q-PCR. Finally, the possible mechanisms of the photoprotective effects of TB were further investigated, focusing on the inflammation-related NF-κB, oxidative stress-related Nrf2, and collagen degradation-related MAPK/AP-1 signaling pathways.

## 2. Materials and Methods

### 2.1. Preparation of Theabrownin (TB)

TB was provided by the Sichuan Provincial Tibetan-tea Industry Engineering Technology Research Center (produced in 2022). The extraction and solution preparation processes of TB were performed using the method outlined by Huang et al. [20]. Ya’an Tibetan tea was steeped at a ratio of 1:50 (*w*/*v*) in boiling water for 40 min. This process was performed twice, and the filtrates from both extractions were combined. The solution was filtered through four layers of gauze and then suction filtered. The obtained liquid was concentrated under reduced pressure at 50–60 °C. The concentrated solution was subsequently cooled to obtain a thick Tibetan tea concentrate. This was extracted with an equal volume of n-butanol (Chengdu Chron Chemical Co., Ltd., Chengdu, China) by shaking for 3 min, and the process was repeated three times. The aqueous phase was then subjected to extraction with an equal amount of chloroform (Chengdu Chron Chemical Co., Ltd., Chengdu, China) three times. The aqueous phase obtained after chloroform extraction was subsequently extracted three times using an equal amount of ethyl acetate (Chengdu Chron Chemical Co., Ltd., Chengdu, China). The final aqueous phase was concentrated under reduced pressure at 50–60 °C until it became viscous. The obtained concentrated solution was vacuum-dried at 70 °C to yield the final powder form. The dosage of TB was determined based on earlier studies on intragastric administration of TB derived from Pu-erh tea [20,21]. The middle dosage was set at 225 mg/kg/day for intragastric administration, with low and high dosages at 75 and 675 mg/kg/day, respectively. TB powder (150, 450, 1350 mg) was dissolved in 2 mL of distilled water for each dosage, filtered using a 0.22 μm disposable needle filter and prepared for administration.

### 2.2. Animal Experiments

#### 2.2.1. Ethical Statement

All experiments in this study were conducted in compliance with the national legislation of China and the guidelines of the Experimental Animal Center at Sichuan Agricultural University (Chengdu, China). Approval for all experimental protocols and research designs was obtained from the Institutional Animal Care and Use Committee of the Experimental Animal Center at Sichuan Agricultural University (Permit No: SICAU-2015-033).

#### 2.2.2. Animals and Experimental Design

Female SPF-level BALB/c hairless mice (6–8 weeks old, weighing 16 ± 2 g) were purchased from Dashuo Experimental Animal Co., Ltd. (Chengdu, China) under production license no. SCXK (Chuan) 2020-030. The mice were housed in standard SPF animal facilities with controlled temperature (22 ± 2 °C), humidity (50–60%), a 12/12 h light/dark cycle, and free access to standard chow and water. After a one-week acclimation period, the mice were randomly divided into six groups (n = 8 per group): (1) normal control group (NC): no UVB irradiation, only physiological saline; (2) UVB-irradiated control group (RC): administered with physiological saline before UVB exposure; (3) positive control group (PC): administered with vitamin C (50 mg·kg^−1^·bw) before UVB exposure; (4) low-dose TB group (TBL): pretreated with low-dose TB (75 mg·kg^−1^·bw) before UVB exposure; (5) medium-dose TB group (TBM): pretreated with medium-dose TB (225 mg·kg^−1^·bw) before UVB exposure; (6) high-dose TB group (TBH): pretreated with high-dose TB (675 mg·kg^−1^·bw) before UVB exposure. All mice were orally gavaged with a volume of 0.1 mL/day, and the dosage was adjusted weekly based on their body weight for 14 days. Mice in all groups, except the NC group, underwent UVB irradiation following a standardized protocol.

#### 2.2.3. UVB Procedure and Construction of Photodamage Model

The minimum erythema dose (MED), an internationally recognized modeling method, was used to construct the skin photodamage model [31,32]. An array of two UVB lamps (20 W, 320 nm) was installed in parallel at the top of a UVB light box (67 × 47 × 42 cm^3^ in length, width and height, respectively) as the designed apparatus for irradiation in this study, and the irradiation height was set at 30 cm. The UVB lamps were preheated for 10 min before UVB irradiation, and after 30 min of administration, the backs of mice were exposed to UVB radiation once a day. The MED was determined in preliminary experiments to be 120 mJ/cm^2^. On the first day, mice in each group, except for the normal control group, received 1 MED light dose for 20 min, and the light dose was increased by 0. 5 MED every two days until reaching a cumulative dose of 4.2 J/cm^2^ by the 14th day. Correspondingly, the UVB exposure time also increased by 10 min every two days. The mice were anesthetized with ether to induce coma at the end of the experiment. Blood was collected via the eye lid, and the mice were euthanized using cervical dislocation. The dorsal skin was photographed, removed, fixed with 4% paraformaldehyde (Chengdu Chron Chemical Co., Ltd., Chengdu, China) for histological analysis, and then frozen in liquid nitrogen before being stored at −80 °C for subsequent ELISA, q-PCR, and Western blotting tests.

#### 2.2.4. Morphological and Histological Examination

Daily observations were conducted on the mental state and dorsal skin of mice. On the final day, 35 volunteers from the animal medical college of Sichuan agricultural university scored the degree of skin photodamage on the backs of mice according to the scoring criteria (Table 1) [33,34,35]. The average macroscopic score for mouse dorsal skin was calculated, where higher scores indicated more severe skin damage.

Skin tissues from the backs of mice were removed, flattened after removing subcutaneous fat, fixed in 4% paraformaldehyde for more than 24 h, dehydrated in a gradient ethanol solution, made translucent in xylene (Chengdu Chron Chemical Co., Ltd., Chengdu, China), and embedded in paraffin. The sections were prepared into approximately 4 μm thick slices using a Leica rotary microtome. These section slides were stained with H&E (Wuhan Servicebio Co., Ltd., Wuhan, China) to assess skin changes, while Masson’s trichrome staining (Wuhan Servicebio Co., Ltd., Wuhan, China) was used to analyze collagen fiber content and arrangement alterations. Six different areas were randomly selected from the epidermis and dermis of each section for evaluation and quantification using an optical microscope.

#### 2.2.5. Determination of SOD and MDA

A 0.2 g sample of skin tissue was homogenized in 9x (% *V*/*V*) deionized water and centrifuged at 12,000 rpm for 5 min. The collected supernatant and 20 μL of serum was used for the determination of SOD activity, and the level of MDA in blood and skin tissue were determined according to the instructions provided by Jiangsu Jincheng Bioengineering Research Institute (Wuxi, China).

#### 2.2.6. Determination of Photodamage-Related Proteins in Skin Tissue by ELISA

ELISA kits were used to detect the contents of MMP-1, IL-1β, IL-6, and TNF-α in skin tissue samples. The specific steps were strictly performed according to the instructions provided in the kit (Jiangsu Meimian Industrial Co., Ltd., Yancheng, China).

#### 2.2.7. q-PCR Analysis

The transcript levels of p38 MAPK, ERK, AP-1, NF-κB, Nrf2, and Caspase-3 mRNA were determined to understand the protective mechanism of TB against UVB-induced skin damage. RNA was extracted from mouse skin using the RNAiso Plus kit (Takara Bio Inc., Beijing, China). Reverse transcription was performed following the manufacturer’s instructions. Primers for the target genes were designed and validated (Table 2). The PCR reaction system temperature was set to 94 °C for 30 s, followed by 40 cycles at 94 °C for 15 s, and fluorescence data were collected at 60 °C.

#### 2.2.8. Western Blotting Analysis

Western blotting was further conducted to analyze the expression levels of the proteins (p38 MAPK, ERK, NF-κB, Nrf2) related to antioxidant capacity, inflammatory response, and skin collagen fibers. Skin tissue samples were lysed in RIPA buffer and subsequently centrifuged at 12,000 rpm for 15 min. The resulting supernatant was employed in protein determination. Proteins were separated via 8% SDS-PAGE gel electrophoresis and transferred to a PVDF membrane. The membrane was blocked in 5% BSA solution and subsequently incubated with primary antibodies (dilution 1:500) against p38 MAPK, ERK, NF-κB, and Nrf2 overnight at 4 °C. After washing, secondary antibodies (dilution 1:2000) were added for incubation at room temperature for 1.5 h. ECL detection reagent was used as the substrate, and images were captured using chemiluminescence imagery. Protein expression was quantified using ChemiScope 2.6.1.0 Analysis software.

#### 2.2.9. Statistics Analysis

The results were presented as mean ± standard deviation (SD). ‘n’ represents the number of animals/group, and representative images are shown herein. Univariate analysis of variance was conducted using DPS V9.01, and the LSD method was applied for multiple comparisons. A *p*-value of less than 0.05 (*p* < 0.05) was considered statistically significant.

## 3. Results and Discussion

### 3.1. Effects of TB on the Macroscopic Appearance of Dorsal Skin in UVB-Irradiated Mice

**Figure 2:**
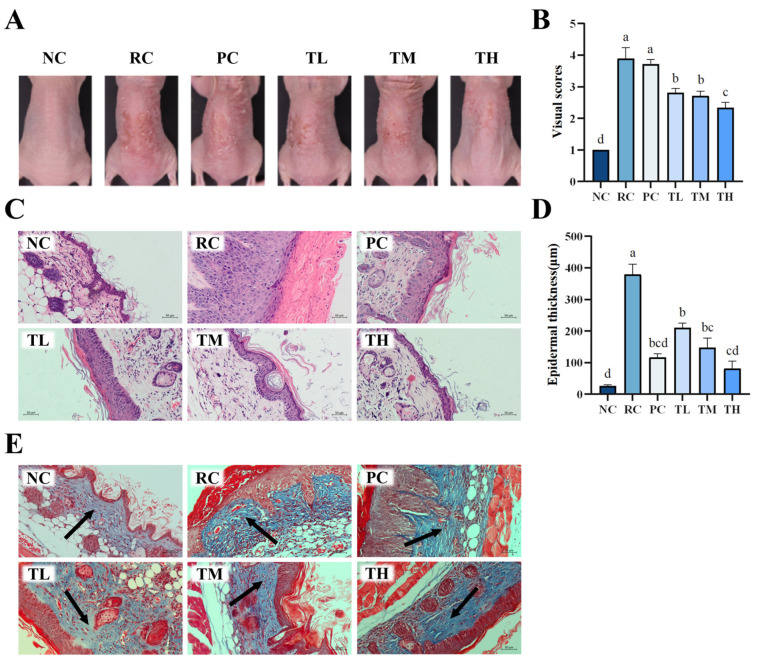
Effects of theabrownin on UVB-induced skin photodamage in BALB/c mice. (**A**) Visual characteristics of the dorsal skin at the completion of the experiment under various treatment conditions. (**B**) Macro evaluation scores of dorsal skin in the different groups (n = 8). (**C**) Histological images of H and E staining of dorsal skin sections. (**D**) Measurement of epidermal thickness. (**E**) Images of Masson’s trichrome-stained dorsal skin sections. Slices stained with dark blue indicate collagen fiber (arrow point), red indicates cytoplasm and muscle fiber. The scale bars for H&E staining and Masson staining are both 50 μm, 200×. Bars, mean ± SEM. Data are presented as mean ± standard deviation (n = 8). Statistical analyses were conducted using DPS V9.01 software with ANOVA (post hoc LSD), and significance was determined at *p* < 0.05. The letters abcd in Figure 2B,D stand for the significance of differences between groups. Note: NC: normal control group; RC: radiation control group; PC: positive control group; TL: low-dose group of theabrownin; TM: medium-dose group of theabrownin; TH: high-dose group of theabrownin.

In recent years, macroscopic scoring methods have gained popularity in evaluating the efficacy of drugs related to photodamage. To assess the extent of UVB-induced damage to mouse skin and the protective effects of TB regarding skin photodamage, a macroscopic scoring method was employed. As shown in Figure 2A, the dorsal skin of mice in the normal control group exhibited smooth texture, even coloration, and overall healthy skin condition. Conversely, mice in the UVB radiation control group showed distinct signs of skin photodamage, including dryness, roughness, peeling, prominent red patches, and inflammation after 2 weeks of UVB irradiation, indicating the successful modeling of UVB-induced skin damage. After the pretreatment of TB, the adverse appearances of dorsal skin caused by UVB irradiation were significantly improved, and the effects of which were even better than the positive control group (vitamin C). The preventive effect of TB was dose-dependent, with the high-dose group displaying minimal peeling, and the suppression of redness and inflammation being most pronounced, nearly resembling that of the normal group. Additionally, macroscopic scores of the dorsal skin in the radiation control group were notably higher than those observed in the normal control group (Figure 2B). In contrast, the macroscopic scores of dorsal skin in all TB-pretreated groups were significantly lower than those in the radiation control group, with the high-dose group scoring the lowest, indicating the least skin photodamage. These findings were consistent with the observed skin appearance, suggesting that TB effectively prevents UVB-induced skin photodamage in a dose-dependent manner.

### 3.2. Effects of TB on the Histochemical Damages of Dorsal Skin in UVB-Irradiated Mice

To further confirm the protective effects of TB on UVB-induced skin damage, Hematoxylin and Eosin (HE) staining and Masson staining were performed to evaluate the structure of skin tissue. The results showed that the skin tissue structure of mice in the normal control group was intact, with regular cell arrangement, distinct epidermal papillae, and a thin, elastic and evenly distributed dermis with tightly arranged collagen fibers. After 14 days of continuous UVB radiation, the skin of mice in the radiation control group exhibited typical signs of photodamage, including abnormal epidermal hyperplasia, significant thickening, disappearance of dermal papillae, extensive rupture of collagen fibers in the dermis, twisting and deformation, and pronounced infiltration of inflammatory cells (Figure 2C,E). In contrast, the photodamage in the dorsal skin of mice pretreated with TB was attenuated in a dose-dependent manner. The high-dose group exhibited a thinner epidermis, prominent dermal papillae, a marked reduction in the rupture of collagen fiber bundles in the dermis, and more orderly arrangement, which was superior to the positive control group and even approaching that of the normal control group.

Epidermal thickness is a quantitative parameter for assessing skin photodamage and inflammation [36]. The measurement results for the thickness of epidermis of mouse dorsal skin are displayed in Figure 2D. UVB radiation led to a significant increase in epidermal thickness (*p* < 0.01), with the thickness rising from 26.37 µm in the normal control group to 379.41 µm in the UVB radiation control group, representing a 14.39-fold increase. Interestingly, the epidermal thickness of mice in the TB-pretreated groups showed a dose-dependent decrease. High-dose TB could significantly improve the excessive keratinization of the skin’s stratum corneum and inflammatory infiltration, even surpassing the positive control group. The macroscopic observations and histopathological results collectively suggest that TB pretreatment can effectively protect against UVB-induced skin photodamage.

### 3.3. Effects of TB on the Antioxidant Capacity of UVB-Irradiated Mice

Excessive UVB radiation and the resulting increase in reactive oxygen species (ROS) lead to a decrease in endogenous antioxidant capacity and oxidative stress damage in cellular structures, a crucial mechanism in the pathogenesis of skin photodamage [37]. The Nrf2 signaling pathway is considered as one of the most important antioxidant pathways; its activation can upregulate the expression of various antioxidant proteins and phase II detoxifying enzymes downstream, enhancing the ability of cells to eliminate reactive oxygen species (ROS) and alleviating oxidative stress-related damage [38,39]. To evaluate the antioxidant capacity of TB, the expression of Nrf2, an important transcription factor, and its downstream antioxidant enzyme SOD were determined along with the oxidative product MDA of biomolecules in skin tissue and serum.

**Figure 3:**
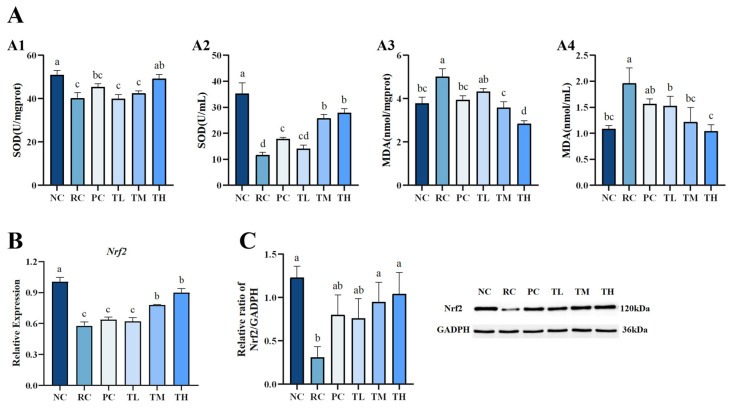
Effects of theabrownin on antioxidant capacity of mice. (**A**) The antioxidant activity of theabrownin. (**A1**) SOD activities in the skin. (**A2**) SOD activities in the serum. (**A3**) MDA content in the skin. (**A4**) MDA content in the serum. (**B**) Gene expression levels of Nrf2. (**C**) Protein expression levels of Nrf2 (n = 8). Proteins were visualized by Western blotting. Data are presented as mean ± standard deviation (n = 8). Statistical analyses were performed using DPS V9.01 software with ANOVA (post hoc LSD), and significance was determined at *p* < 0.05. The letters abcd in Figure 3 stand for the significance of differences between groups.

As shown in Figure 3, UVB suppressed the Nrf2 pathway and led to a significant decrease in Nrf2 mRNA and protein expression levels (Figure 3B,C), as well as a decline in the activities of SOD, accompanied by a notable increase in the oxidative biomarker MDA content (Figure 3A). TB pretreatment prevented these adverse effects by upregulating the expression levels of Nrf2 mRNA and protein, subsequently enhancing SOD enzyme activities and reducing UVB-induced damage to biomolecules, leading to a significant decrease in MDA content. The expression levels of Nrf2 mRNA and protein in the skin tissue of the high-dose group were significantly elevated by 55.42% and 237.12%, respectively, compared to the radiation control group (*p* < 0.01), as shown in Figure 3B,C. Meanwhile, SOD enzyme activity in the serum of the high-dose group increased by 139.91% (*p* < 0.01) while MDA content decreased by 46.77% (*p* < 0.05), relative to the radiation control group, as depicted in Figure 3A2,A4. The antioxidant capacity of tea is considered one of its most important health benefits, which is closely related to its biochemical components, such as tea polyphenols and their oxidation products, tea polysaccharides, and flavones [40,41]. Among them, tea polyphenols and their oxidation products are considered the most critical factors influencing the antioxidant capacity of tea [14,42]. Our previous studies have reported that TB has outstanding protective effects on the antioxidant and hematopoietic systems of rats damaged by ^60^Coγ radiation. TB significantly enhanced the antioxidant capacity of SOD, CAT, T-SOD, and T-AOC in serum and liver tissue and bone marrow DNA content of the femur in the mice in a dose-dependent manner [25]. In addition, a recent study revealed that TB improved the liver oxidative stress (GSH-Px, SOD, and MDA) in aging mice [14]. In this study, we observed that TB had a potent antioxidant effect and could significantly reduce oxidative stress-related damage. The associated mechanism may involve the activation of the Nrf2 signaling pathway, inducing Nrf2 nuclear translocation and subsequently enhancing the activities of antioxidant enzyme SOD to protect cell membrane lipids from free radical attacks, reinforcing their role in resisting oxidative stress damage.

### 3.4. Effects of TB on the Inflammatory Response Triggered by UVB Irradiation

The aforementioned macroscopic observations revealed erythema (red patches), dandruff (scales), and inflammatory infiltration on the dorsal skin of mice, indicating an inflammatory cell infiltration-type reaction caused by excessive ultraviolet (UV) radiation exposure [43]. UVB exposure stimulates the generation of reactive oxygen species (ROS), activating the MAPK pathway to promote the expression of matrix metalloproteinase-1 (MMP-1) and leading to inflammatory cell infiltration that leads to collagen fiber degradation. Additionally, ROS activate the NF-κB signaling pathway in mouse skin. NF-κB, a protein complex composed of p65 and p50 subunits, functions as a universal transcription factor that regulates the expression of numerous inflammatory cytokines associated with the inflammatory response. Reactive oxygen species (ROS) can induce NF-κB release from its inhibitor I-κB, enabling the translocation of active NF-κB to the nucleus to activate pro-inflammatory cytokines such as IL-1β, IL-6, and TNF-α, thereby exacerbating the inflammatory process and leading to skin erythema and inflammation [44].

**Figure 4:**
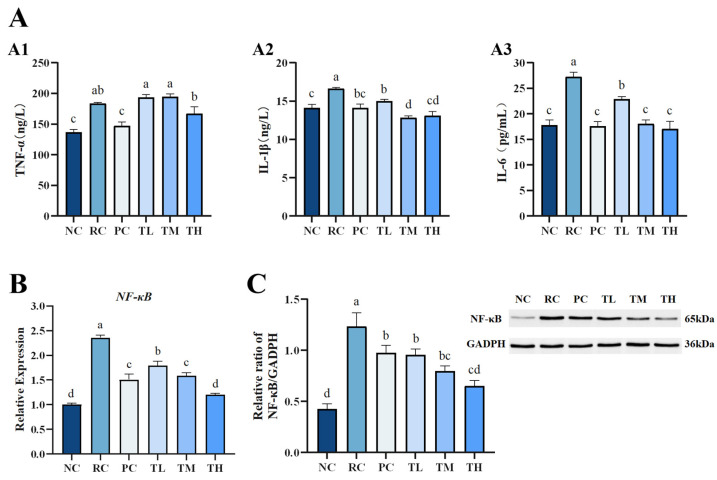
Effects of theabrownin on the inflammatory pathway in the skin. (**A**) Levels of inflammatory proteins, including TNF-α (**A1**), IL-1β (**A2**), and IL-6 (**A3**). (**B**) Expression of NF-κB at the mRNA level. (**C**) Expression of NF-κB at the protein level (n = 8). Proteins were visualized by Western blotting. Data are presented as mean ± standard deviation (n = 8). Statistical analyses were conducted using DPS V9.01 software with ANOVA (post hoc LSD), and significance was determined at *p* < 0.05. The letters abcd in Figure 4 stand for the significance of differences between groups.

The study assessed skin inflammation by evaluating key inflammatory factors, including TNF-α, IL-1β, IL-6, and by assessing NF-κB mRNA and protein expression levels, which are critical mediators of the inflammatory signal transduction pathway. We found that UVB radiation significantly upregulated the levels of TNF-α, IL-1β, and IL-6 in mouse skin tissues (Figure 4A). Following TB pretreatment, there was a slight decrease in TNF-α levels (Figure 4A1), and a significant or highly significant reduction in IL-1β and IL-6 levels (Figure 4A2,A3). This suggests that TB pretreatment could inhibit the inflammatory response mainly by regulating IL-1β and IL-6; hence, TNF-α is possibly not the primary pathway for TB to modulate the inflammatory response. Further investigation into the nuclear transcription factor NF-κB revealed a significant increase in phosphorylated NF-κB levels in mouse skin after UVB radiation (Figure 4B,C). The expression levels of NF-κB mRNA and protein in the skin of the radiation control group were 1.35-fold and 1.89-fold higher than those in the normal control group, respectively, indicating UVB-induced overexpression of the NF-κB pathway. In contrast, all TB-pretreated groups showed significantly reduced phosphorylation of NF-κB, resulting in a 49.03% and 47.03% decrease in NF-κB mRNA and protein levels, respectively. Moreover, the inhibitory effect of the high-dose TB group was superior to the positive control group and close to the normal control group. The results suggest that IL-1β and IL-6 are potentially the primary effective targets for TB to regulate the inflammatory response, which is similar to findings of previous studies that TB in Pu-erh tea ameliorated inflammation via inhibiting the level of IL-1β and IL-6 in the NF-κB signaling pathway [14,45]. It is noteworthy that TB in this study has less significant effects on the TNF-α, but TB in Pu-erh tea significantly decreased the level of TNF-α, which might be due to the apparent differences in the composition of between Ya’an Tibetan tea and Pu-erh tea. Additionally, differences in processing techniques and regional characteristics could also contribute to these discrepancies. Considering the formation of erythema on the dorsal skin of mice, it is speculated that TB may produce a good anti-inflammatory effect by downregulating the expression of pro-inflammatory cytokines IL-1β and IL-6, as well as interrupting the NF-κB inflammatory pathway.

### 3.5. Effects of TB on Collagenase and Its Signaling Pathway in Photodamaged Mouse Skin

Masson’s staining of skin revealed that UVB induces collagen degradation in the dermal layer and the distortion of elastic fibers, mainly due to the excessive ROS produced by UVB radiation. ROS, acting as secondary messengers in the skin, triggers the Mitogen-Activated Protein Kinase (MAPK) family, mainly including proline-directed Ser/Thr kinases comprising extracellular signal-regulated kinases (ERKs), p38, and c-Jun NH2-terminal kinase (JNK). The phosphorylation of ERK induces the activation of c-Fos, while the activation of p38 and JNK induces the expression of c-Jun. The combination of c-Jun and c-Fos forms the transcription factor AP-1, which in turn promotes the overproduction of matrix metalloproteinase-1 (MMP-1). This excessive synthesis of MMP-1 results in the degradation of collagen [46,47].

**Figure 5:**
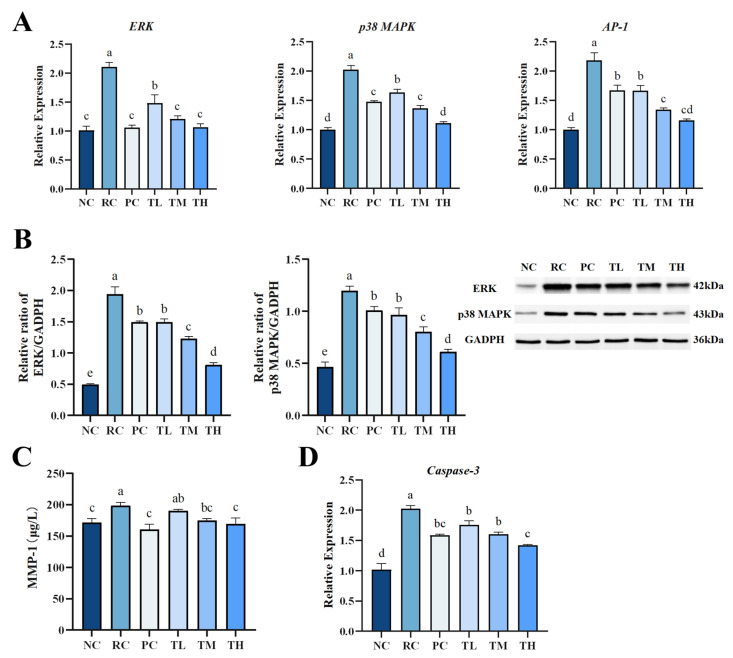
Theabrownin significantly suppressed the hyperactivation of the MAPK and Caspase-3 pathways in photodamaged skin. (**A**) Gene expression levels of ERK, p38 MAPK and AP-1. (**B**) Protein expression levels of ERK, and p38 MAPK. (**C**) Protein levels of MMP-1 in skin. (**D**) Gene expression levels of Caspase-3 gene (n = 8). Statistical analyses were performed using DPS V9.01 software with ANOVA (post hoc LSD), and significance was determined at *p* < 0.05. The letters abcde in Figure 5 stand for the significance of differences between groups.

MMP-1 is the first proteinase that triggers collagen degradation, breaking down type I collagen fibers. Type I collagen fibers are a major extracellular matrix component providing structural support to the skin; hence, their breakdown leads to the destruction of the extracellular matrix structure, resulting in dermal decomposition and typical photodamage symptoms, such as delayed wound healing, decreased elasticity, and roughness [48,49,50]. Masson’s staining demonstrated that TB effectively reversed the UVB-induced degradation of collagen and distortion of elastic fibers in the dermal layer of the skin. In this study, interstitial collagenase (MMP-1) and its upstream MAPK/AP-1 signaling pathway were selected to further investigate the protective effect of TB on skin collagen.

q-PCR and Western blot analysis revealed that the MAPK and AP-1 signaling pathways were activated after 2 weeks of UVB exposure. The mRNA expression levels of p38 MAPK, ERK, and AP-1 were upregulated by 1.02-fold, 1.09 times, and 1.18 times, respectively (Figure 5A), and the protein expression levels of p38 MAPK and ERK increased by 1.56 times and 2.91 times, respectively (Figure 5B), compared to the normal control group. Interestingly, the upregulated p38 MAPK, ERK, and AP-1 mRNA and protein in the skin of all TB-pretreated groups were dramatically inhibited in a dose-dependent manner (*p* < 0.01). The effects of TB pretreatment in middle and high doses were comparable to or better than those of the positive control group (*p* < 0.01), while the high-dose group showed expression levels close to the normal control group. As exhibited in Figure 5C, UVB radiation significantly increased the synthesis of MMP-1 protein in mouse skin, which is in agreement with published data that UVB upregulates MMP levels [48]. However, TB pretreatment significantly lowered the MMP-1 content. Although low-dose TB had a less noticeable effect, the middle- and high-dose TB groups had significantly lower MMP-1 levels than the radiation control group, comparable to the normal control group. Additionally, recent evidence suggests that tea polyphenols, such as catechins, and their oxidation products can reduce MMP-1 levels by regulating the expression of ERK, p38 MAPK and NF-κB in the MAPK pathway, preventing collagen degradation [41,51]. TB is an oxidation product of tea polyphenols and thus retains some of their properties. Therefore, it is speculated that the mechanism by which TB prevents UVB induced skin collagen degradation is similar to that of tea polyphenols. Collectively, these results indicate that TB pretreatment effectively protects against the degradation and distortion of collagen in the skin, which is likely associated with inhibiting MMP-1 synthesis and the phosphorylation of p38 MAPK, ERK, and AP-1 in the MAPK pathway.

### 3.6. Effects of TB on the Gene Expression of Apoptosis Effector Protein Caspase-3 in Photodamaged Mouse Skin

Excessive oxidative stress and inflammation not only lead to collagen degradation but also induce cell apoptosis, a cellular response to damage [52]. Studies have shown that exposure to UVB radiation promotes cell apoptosis by damaging mitochondria and triggering death receptors [53,54]. Caspase-3 is a crucial apoptosis effector protein that can induce cell apoptosis through various pathways, making it the primary executor of apoptosis [55]. Elevated Caspase-3 expression is a major contributor to cell apoptosis. In this study, we further investigated the impact of TB on UVB-induced apoptosis in skin cells by determining the expression of Caspase-3. As shown in Figure 5D, UVB radiation dramatically upregulated the expression of Caspase-3, with a significant increase (99%) in Caspase-3 mRNA levels in the skin of the radiation control group compared to the normal control group. However, UVB-induced Caspase-3 mRNA increase could be significantly and dose-dependently surpassed by all doses of TB. This is consistent with the results of Ricássio’s study on epigallocatechin, where it was evident that epigallocatechin could reduce cell apoptosis by suppressing the gene expression of Caspase-3 through the caspase signaling cascade [56].

### 3.7. Protective Mechanisms of TB in Photodamaged Mouse Skin

**Figure 6:**
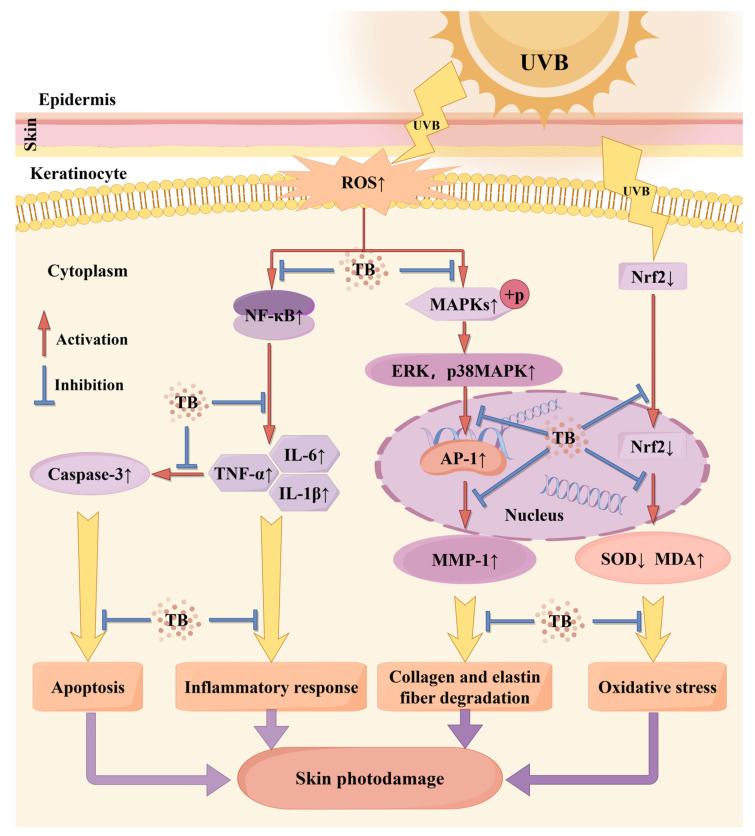
Possible mechanism of theabrownin inhibition of UVB-induced skin photodamage. ↑: level was increased; ↓: level was decreased. Blue color represents effective. Note: by Figdraw.

Our research is the first to reveal that theabrownin (TB) derived from Ya’an Tibetan tea possesses significant photoprotective properties. This study offers valuable preclinical evidence supporting the potential use of TB as a natural agent to prevent skin photodamage and enhance skin health in the future. The potential mechanisms underlying UVB-induced skin damage in Balb/c mice are illustrated in Figure 6, based on the KEGG PATHWAY Database. Firstly, UVB radiation inhibited the antioxidant response element-Nrf2 signaling pathway and lead to decreased activities of the antioxidant enzyme SOD, increasing oxidative stress and MDA levels on biomolecules. Conversely, TB pretreatment upregulated Nrf2 expression, while increasing SOD activities and decreasing MDA levels in mice skin, which partly explains the activation in Nrf2 signaling pathway seen in TB-pretreated mice. It is well known that UVB radiation induces the production of ROS. On the one hand, ROS can trigger the phosphorylation of the Mitogen-Activated Protein Kinase (MAPK), and activate ERK, p38 MAPK and AP-1, thereby promoting synthesis of MMP-1, which mainly affects collagen and elastin fiber degradation. Instead, TB pretreatment reversed all these trends induced by UVB, which suggested that TB regulated the MAPK signaling pathway. On the other hand, excessive accumulation of ROS activated NF-κB, which further stimulated the production of inflammatory cytokines that are involved in the inflammatory response. TB pretreatment reduced the over-production of inflammatory factors (IL-1β, IL-6, and TNF-α) by inhibiting NF-κB signaling pathway, thereby alleviating inflammation induced by UVB. Also, another significant finding in this present study was the finding that the level of Caspase-3 mRNA was significantly reduced when UVB-irradiated mice were pretreated with TB. This decrease in Caspase-3 may mean a reduction in ROS-induced cell apoptosis, which may be associated with activation of the NF-κB pathway. Recent studies reported that NF-κB activation induces senescence, and stimulates the inflammatory and apoptotic pathways, and increased the inflammatory cytokines levels of IL-6, IL-1β secretion, and then facilitates Caspase-3 transcription, leading to activation of caspase dependent apoptosis [57,58]. Further research is needed on the specific signaling pathway of Caspase-3 regulating cell apoptosis. In summary, the preventive effect of TB may be associated with downregulation of the MAPK/NF-κB signaling pathway, and promotion of the Nrf2 signaling pathway in the skin. Nevertheless, given that photodamage is a complex process, further studies should be conducted to determine whether there are other signaling pathways involved in the process of anti-photodamaging by TB.

## 4. Conclusions

In conclusion, our study suggests that TB significantly inhibits UVB-induced skin photodamage in BALB/c mice. This protective effect appears to be related to the attenuation of oxidative stress via activation of the Nrf2 signaling pathway, as well as a decrease in inflammatory response, cell apoptosis, and collagen degradation through the inhibition of the MAPK/NF-κB signaling pathway. This study provides robust scientific evidence supporting the potential of TB as a preventive agent against UVB-induced skin photodamage, elucidating its mechanisms across multiple dimensions, including physiological, biochemical, genetic, and proteomic aspects. To the best of our knowledge, this study is the first to clearly demonstrate the prominent photoprotective effects of TB from Ya’an Tibetan tea using data from a hairless photodamage mouse model.

## Figures and Tables

**Table 1:** Evaluation criteria for skin photodamage.

Score	Skin Condition
1.0	Skin appears flesh-colored, smooth and plump
2.0	Skin appears flesh-colored and slightly rough
3.0	Erythema, moderate roughness, and deepening of skin lines
4.0	Erythema, obvious roughness, slight peeling, and wrinkles on the skin
5.0	Purplish red and ulcerative state of the skin, which is rough and thickened, with significant desquamation and increased wrinkles

**Table 2:** Gene sequence of primers.

Gene	Direction	Primer Sequence
NF-κB	Forward	5′-GGA GGC ATG TTC GGT AGT GG-3′
	Reverse	5′-CCC TGC GTT GGA TTT CGT G-3′
ERK	Forward	5′-CAC TGG CTT TCT GAC GGA GT-3′
	Reverse	5′-CCG GTT GGA GAG CAT CTC AG-3′
p38 MAPK	Forward	5′-AGC CAA TTC CAG TGT TGG AC-3′
	Reverse	5′-TTC TGG GCT CCA AAT GAT TC-3′
AP-1	Forward	5′-ACG ACC TTC TAC GAC GAT GC-3′
	Reverse	5′-GCC AGG TTC AAG GTC ATG CT-3′
Caspase-3	Forward	5′-GAG CTT GGA ACG GTA CGC TAA-3′
	Reverse	5′-CCA CTG ACT TGC TCC CAT GT-3′
β-action	Forward	5′-GCT GTG CTA TGT TGC TCT AG-3′
	Reverse	5′-CGC TCG TTG CCA ATA GTG-3′

## Data Availability

The original contributions presented in the study are included in the article, further inquiries can be directed to the corresponding authors.
